# Kawasaki Disease Recurrence as a Diagnostic and Therapeutic Challenge: A Case Report

**DOI:** 10.7759/cureus.104872

**Published:** 2026-03-08

**Authors:** Irma Martínez Tovar, Lourdes E Escalante Madera, Ana M Tiscareño Guzmán, Karla V Luevano Villa

**Affiliations:** 1 Pediatrics, General Hospital Aguascalientes of the Institute of Security and Social Services of State Workers, Aguascalientes, MEX; 2 Medicine, Autonomus University of Zacatecas, Zacatecas, MEX; 3 Medicine, Universidad Cuauhtémoc Aguascalientes Campus, Aguascalientes, MEX; 4 Medicine, Autonomus University of Guadalajara, Guadalajara, MEX

**Keywords:** aneurysm, inmunoglobulin, kawasaki disease (kd), refractory, vasculitis

## Abstract

Kawasaki disease (KD) is a systemic vasculitis typically affecting children. It is usually self-limited, lasting less than two weeks without treatment; however, cardiovascular complications can occur, causing significant morbidity and mortality. Diagnosis is generally clinical, and treatment follows standardized guidelines, primarily intravenous immunoglobulin (IVIG) combined with acetylsalicylic acid (ASA), with or without corticosteroids, depending on the severity. Among KD types, refractory disease is defined by persistent fever after initial therapy, and recurrent disease is defined by the presence of at least three of the five classic KD criteria occurring at least 14 days after returning to baseline health. This case reports an 11-month-old female patient with recurrent KD without coronary complications after appropriate initial IVIG treatment.

## Introduction

Kawasaki disease (KD), formerly called mucocutaneous lymph node syndrome, is a self-limited systemic vasculitis that primarily affects children under five years of age, especially of Asian descent. Though rare in adults, it remains the leading cause of acquired heart disease in children in developed countries and is second only to IgA vasculitis among pediatric vasculitides [1-6].

The disease affects medium-sized arteries and can involve multiple organs and tissues. While its exact etiology is unknown, genetic predisposition and immune system dysregulation-both innate and adaptive-are implicated [3,7,8].

Diagnosis is clinical and based on the American Heart Association (AHA) 2024 criteria, classifying cases as complete or incomplete. Early recognition-ideally within the first 10 days of fever-is essential to initiate treatment and prevent complications. First-line therapy includes intravenous immunoglobulin (IVIG) and acetylsalicylic acid (ASA) [9,10].

Cardiovascular complications are the most severe, including coronary artery aneurysms that may progress to ischemia, infarction, arrhythmia, or sudden cardiac death if untreated [2,10,11]. Other possible complications include KD shock syndrome, macrophage activation syndrome, sensorineural hearing loss, and involvement of other arterial beds [10,11].

In IVIG-resistant or recurrent cases (occurring in 15-20% and up to 3.6% of patients, respectively), additional interventions such as corticosteroids, infliximab, or a second IVIG dose may be required [12-19].

## Case presentation

An 11-month-old female patient with no relevant medical history presented with a five-day history of fever (39 °C), conjunctival hyperemia, oral mucosal lesions, and a polymorphous rash involving the lower limbs and trunk. Initial outpatient treatment with ibuprofen (10 mg/kg/dose every six to eight hours), phenylephrine, and loratadine (0.1-0.2 mg/kg once daily) was ineffective.

**Figure 1:**
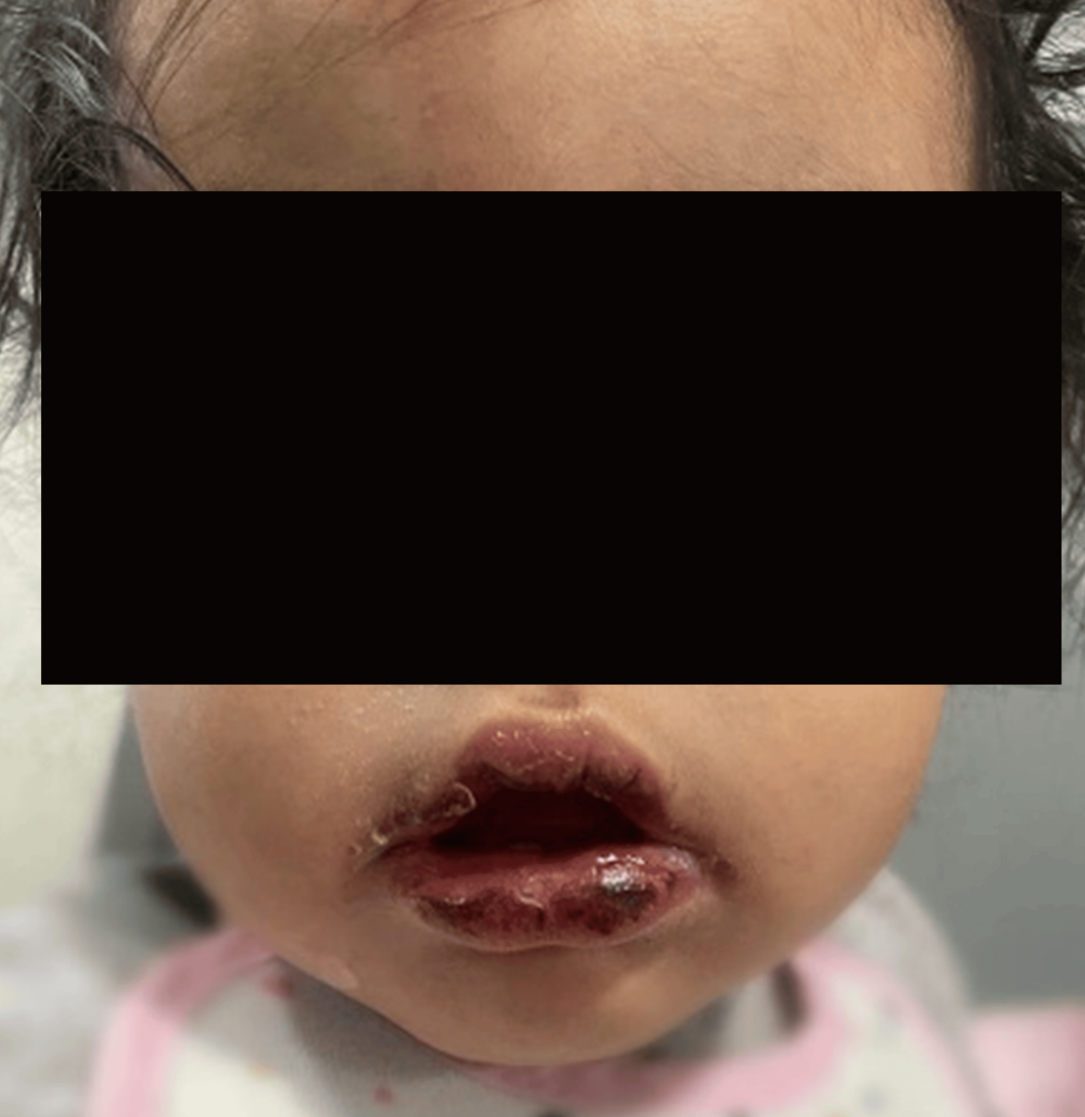
Erythematous oral lesions, fissured lips with mild desquamation, strawberry tongue.

Upon hospital admission, physical examination revealed erythematous oral lesions, fissured lips with mild desquamation, a strawberry tongue, bilateral conjunctival injection, and a polymorphous rash on the trunk and lower extremities (Figure 1).

Initial laboratory studies were obtained (Table 1).

**Table 1:** Laboratory findings during first and second hospitalizations Laboratory results from both hospitalizations. Alterations during the second hospitalization include leukocytosis, neutrophilia, hypoalbuminemia, and elevated liver enzymes. Reference ranges correspond to standard pediatric values for infants under 1 year. The determinations correspond to different clinical moments: hospital admission, after treatment with intravenous immunoglobulin (IVIG), and follow-up during the second hospitalization.

Parameter	Normal range	First hospitalization admission	First hospitalization, post-IVIG	Second hospitalization, admission	Second hospitalization, 24 hours after admission	Second hospitalization, 48 hours after admission
Leukocytes (/µL)	6,000–17,500	8,800	7,600	20,200	13,400	12,600
Lymphocytes (/µL)	4,000–10,000	6,200	4,200	3,560	3,430	4,380
Monocytes (/µL)	200–1,200	400	480	1,490	1,230	1,360
Neutrophils (/µL)	1,000–8,500	1,500	2,400	14,950	8,590	6,520
Hemoglobin (g/dL)	10.5–13.5	12.0	11.4	12.4	11.3	11.8
Hematocrit (%)	33–39	37.0	35.1	38.9	33.4	35.7
Platelets (/µL)	150,000–450,000	450,000	434,000	389,000	339,000	339,000
C-reactive protein (mg/L)	<10	48	>50	>90	59	—
Creatine phosphokinase – CPK (U/L)	38–174	163	—	—	—	—
CPK-MB (U/L)	<25	36	—	—	—	—
Erythrocyte sedimentation rate (mm/hr)	<20	—	10	—	—	12
ALT (U/L) – alanine aminotransferase	<45	51	28	201	109	109
AST (U/L) – aspartate aminotransferase	<50	39	36	236	57	57
Lactate dehydrogenase (U/L)	140–280	278	216	354	216	350
Alkaline phosphatase (U/L)	150–420	208	208	237	213	254
Gamma-glutamyl transferase (U/L)	<40	40	27	111	107	107
Albumin (g/dL)	3.5–5.0	4.1	3.7	3.5	3.3	3.3

The patient was treated with intravenous immunoglobulin (IVIG, 2 g/kg single dose) and acetylsalicylic acid (ASA, 80-100 mg/kg/day divided every six hours) according to current American Heart Association (AHA) guidelines [9], in addition to paracetamol (10-15 mg/kg/dose every six hours) and oral antihistamines.Transthoracic echocardiography demonstrated normal coronary arteries without evidence of ectasia or aneurysms, preserved biventricular systolic function, and normal pulmonary artery systolic pressure. In this case, pro-brain natriuretic peptide (pro-BNP) and N-terminal pro-brain natriuretic peptide (NT-proBNP) measurements were not performed, which is recognized as a limitation, given their potential value for risk stratification. She was discharged with scheduled outpatient follow-up.

**Figure 2:**
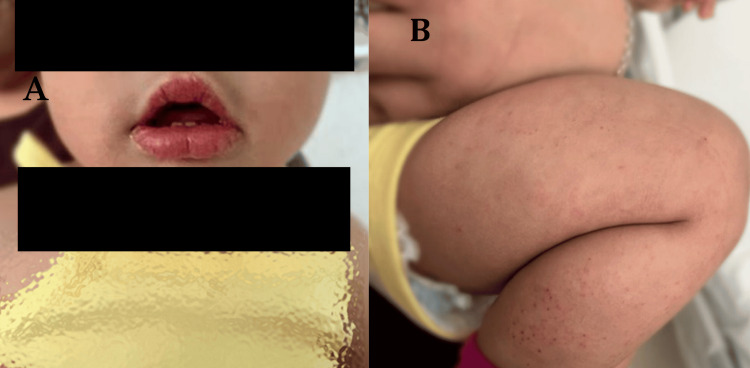
Erythematous lips (A) with mild cracking, truncal and lower limb papular rash (B).

Sixteen days after discharge, the patient was re-evaluated due to recurrence of fever (39 °C), irritability, erythematous lips with mild fissuring, and a papular rash involving the trunk and lower extremities (Figure 2). Follow-up laboratory tests revealed leukocytosis, elevated alanine aminotransferase (ALT), and hypoalbuminemia compared to initial values (Table 1).

She was readmitted and treated with a second dose of IVIG (2 g/kg single dose), ASA (80-100 mg/kg/day), and systemic corticosteroids (methylprednisolone, 2 mg/kg/day intravenously in divided doses). A follow-up transthoracic echocardiogram during the second hospitalization showed no coronary abnormalities, preserved biventricular systolic function, and no evidence of ectasia or aneurysms. The patient became afebrile within 72 hours and was discharged 96 hours later with continued outpatient follow-up. During her clinical course, no periungual desquamation was observed. Infectious causes of fever were actively ruled out through clinical evaluation and laboratory tests. Post-discharge cardiological follow-up was not performed due to the lack of a subcontracted echocardiography service.

## Discussion

KD is a medium-vessel vasculitis of unknown etiology that predominantly affects children and may involve multiple organs and tissues [3,7]. Although self-limited, untreated cases carry a high risk of severe cardiovascular complications, especially coronary artery aneurysms, which can result in myocardial infarction, arrhythmias, or sudden cardiac death [2,10,11]. KD remains the leading cause of acquired heart disease in children in developed countries. In Mexico and Latin America, its epidemiological ranking is less clearly defined due to limited population-based data [4,6].

Diagnosis is clinical and follows the AHA 2024 criteria, classifying cases as complete or incomplete [9]. Early recognition and treatment within the first 10 days of fever are essential to reduce the risk of coronary complications. While echocardiography is not mandatory prior to starting treatment, it should be performed promptly to detect coronary abnormalities and guide follow-up, as recommended by current guidelines [9,10]. In this patient, cardiac biomarkers such as pro-BNP/NT-proBNP were not measured, as they are not routinely available at our institution; therefore, clinical assessment and echocardiographic follow-up were used for risk stratification and monitoring.

Initial treatment consists of a single IVIG infusion combined with high-dose ASA, which is tapered once the patient is afebrile [9,10]. In patients at high risk for IVIG resistance, adjunctive corticosteroid therapy has shown benefit in improving coronary outcomes [12,13].

Refractory KD occurs in 15-20% of cases and is defined as persistent or recurrent fever ≥36 hours after the end of initial IVIG treatment [15,16]. Management includes a second IVIG dose, corticosteroids, or infliximab. In severe, multidrug-refractory cases, cyclosporine or cyclophosphamide may be considered [16].

Recurrent KD, though rare (0.8-3.6%), poses a higher risk of complications. It is defined as a new episode occurring ≥14 days after clinical resolution of the initial episode, with at least three classic clinical features and fever for ≥5 days [17]. These cases may present with more severe manifestations and an increased risk of coronary involvement [18]. Management typically mirrors that of refractory KD, using IVIG, corticosteroids, or biologics such as infliximab [19].

Risk factors associated with IVIG resistance and recurrence include young age, elevated inflammatory markers, hypoalbuminemia, and genetic susceptibility. Epidemiologically, the incidence of KD varies geographically, with lower reported rates in Latin America, likely reflecting underdiagnosis rather than true rarity [20].

Our patient initially presented with a typical KD course, managed according to AHA guidelines [9], with normal initial echocardiography and a favorable clinical response. She was discharged with appropriate follow-up and warning signs. More than two weeks after resolution of the initial episode, she developed fever, conjunctivitis, rash, and oral lesions, fulfilling diagnostic criteria for recurrent KD. She was rehospitalized and treated with IVIG and corticosteroids, showing rapid clinical improvement.

This case highlights the importance of timely recognition of recurrent KD and adherence to established treatment protocols. Early diagnosis and prompt management were crucial in preventing coronary complications and ensuring complete recovery, emphasizing the need for structured follow-up and family education in the long-term care of patients with KD.

## Conclusions

KD remains a significant diagnostic challenge due to its nonspecific clinical presentation and potential overlap with other pediatric conditions. The consequences of delayed recognition can be severe, given the associated risk of coronary artery aneurysms and sudden cardiac death. Clinicians must therefore maintain a high index of suspicion, particularly in febrile children with persistent symptoms, and act promptly to initiate treatment. IVIG continues to be the cornerstone of therapy, effectively reducing the incidence of cardiovascular complications. Nevertheless, awareness of alternative strategies is essential in cases of IVIG resistance or treatment failure, including the use of corticosteroids, biologic agents, or other immunomodulatory therapies. Ultimately, early diagnosis, timely intervention, and individualized treatment approaches are critical to improving outcomes and minimizing the long-term burden of KD.

This case highlights the diagnostic challenges of recurrent Kawasaki disease and underscores the importance of careful clinical assessment and exclusion of alternative causes of fever. As a single case report, it is limited by the absence of certain biomarkers and cannot establish causality. Nevertheless, early recognition, appropriate treatment, and structured follow-up were essential to prevent coronary complications.
